# A Whole-Genome Scan Revealed Genomic Features and Selection Footprints of Mengshan Cattle

**DOI:** 10.3390/genes15091113

**Published:** 2024-08-23

**Authors:** Haijian Cheng, Yang Lyu, Ziao Liu, Chuanqing Li, Kaixing Qu, Shuang Li, Zulfiqar Ahmed, Weidong Ma, Xingshan Qi, Ningbo Chen, Chuzhao Lei

**Affiliations:** 1 Key Laboratory of Animal Genetics, Breeding and Reproduction of Shaanxi Province, College of Animal Science and Technology, Northwest A&F University, Yangling 712100, China; 98061107@163.com (H.C.); 15134378707@163.com (Y.L.); l907117408@163.com (Z.L.); lichuanqing2022@126.com (C.L.); lishuang0407@163.com (S.L.); ningboch@126.com (N.C.); 2 Shandong Key Lab of Animal Disease Control and Breeding, Institute of Animal Science and Veterinary Medicine, Shandong Academy of Agricultural Sciences, Jinan 250100, China; 3 Academy of Science and Technology, Chuxiong Normal University, Chuxiong 675099, China; kaixqu@163.com; 4 Department of Livestock and Poultry Production, Faculty of Veterinary and Animal Sciences, University of Poonch Rawalakot, Rawalakot 12350, Pakistan; zulfiqarahmed@upr.edu.pk; 5 Shaanxi Province Agriculture & Husbandry Breeding Farm, Baoji 722203, China; 13689271488@163.com; 6 Animal Husbandry Bureau in Biyang County, Zhumadian 463700, China; q13938344182@163.com

**Keywords:** Mengshan cattle, genetic diversity, selective signature, genomic research

## Abstract

(1) Background: Mengshan cattle from the Yimeng mountainous region in China stand out as a unique genetic resource, known for their adaptive traits and environmental resilience. However, these cattle are currently endangered and comprehensive genomic characterization remains largely unexplored. This study aims to address this gap by investigating the genomic features and selection signals in Mengshan cattle. (2) Methods: Utilizing whole-genome resequencing data from 122 cattle, including 37 newly sequenced Mengshan cattle, we investigated population structure, genetic diversity, and selection signals. (3) Results: Our analyses revealed that current Mengshan cattle primarily exhibit European taurine cattle ancestry, with distinct genetic characteristics indicative of adaptive traits. We identified candidate genes associated with immune response, growth traits, meat quality, and neurodevelopment, shedding light on the genomic features underlying the unique attributes of Mengshan cattle. Enrichment analysis highlighted pathways related to insulin secretion, calcium signaling, and dopamine synapse, further elucidating the genetic basis of their phenotypic traits. (4) Conclusions: Our results provide valuable insights for further research and conservation efforts aimed at preserving this endangered genetic resource. This study enhances the understanding of population genetics and underscores the importance of genomic research in informing genetic resources and conservation initiatives for indigenous cattle breeds.

## 1. Introduction

Cattle hold significant economic value and occupy a prominent position among domesticated species. Human migration has led to an increasing number of cattle breeds adapted to diverse needs such as milk, meat, leather, and agricultural purposes, resulting in various commercial and local breeds [1]. In recent years, whole-genome sequencing technology has been extensively applied to investigate population structures, genetic diversity, and selection features in livestock. Studies utilizing whole-genome resequencing have revealed distinct local characteristics and environmental adaptability in indigenous cattle populations [2,3,4,5]. Additionally, this technology facilitates the identification of economically important adaptive genes in local cattle breeds [6,7,8]. These insights are crucial for genetic improvement in livestock and the conservation of local breeds.

Mengshan cattle, primarily found in the Yimeng Mountain region, represent one of China’s fifty-seven local cattle breeds and are among the three major local breeds in Shandong Province, alongside Luxi and Bohai Black cattle. The Mengshan cattle are small in stature but strong, an excellent local breed that has been cultivated by the Yimeng people over a long period to adapt to mountainous natural conditions [9]. They are highly favored by the local population as draft animals. With the development of agriculture, draft cattle have gradually been replaced by agricultural machinery. Having fulfilled their historical role, the Mengshan cattle have transitioned from draft animals to a beef breed, making them highly practical, long-lived, genetically stable, docile in temperament, and renowned for their high-quality meat [10].

Mengshan cattle serve as a historical testament to China’s ancient agricultural civilization, holding significant cultural value and being regarded as a heritage breed. Due to weak conservation awareness in the early stages, a large number of Mengshan cattle and commercial cattle cross-bred, thus ignoring the protection of native breed genetic resources [10]. Currently, this breed is classified as a genetic resource on the verge of extinction by national authorities [9]. Therefore, we should attach great importance to the protection of Mengshan cattle, recognizing their cultural and agricultural significance. It is crucial to vigorously improve the breeding and enhancement process of Mengshan cattle to preserve their unique genetic traits, ensure their continued adaptation to local environments, and contribute to the sustainable development of the region’s livestock industry. Based on whole-genome resequencing techniques, Luxi cattle [11] and Bohai Black cattle [12,13] have been reported successively, elucidating their population structure and genetic diversity, as well as the selected candidate regions. However, there is still a lack of research on the population genetics of existing Mengshan cattle, necessitating a more in-depth analysis of their genomic characteristics.

Herein, we utilized whole-genome resequencing data from 122 cattle, including newly sequenced data from 37 Mengshan cattle, to investigate population structure, genetic diversity, and selection signals. By integrating these genomic datasets, our research aims to enhance the genomic information available for Mengshan cattle and deepen our understanding of their genetic characteristics. Specifically, our study seeks to identify genomic regions that have undergone positive selection, potentially linked to adaptive traits beneficial for the survival and productivity of Mengshan cattle in their native environments. By pinpointing these positively selected regions, we hope to uncover key genomic features that contribute to the breed’s unique adaptations and resilience. These findings are expected to offer valuable insights into the genetic basis of adaptive traits in Mengshan cattle and to inform future research and conservation efforts for this endangered genetic resource.

## 2. Materials and Methods

### 2.1. Sample Preparation and DNA Resequencing Data

**Figure 1:**
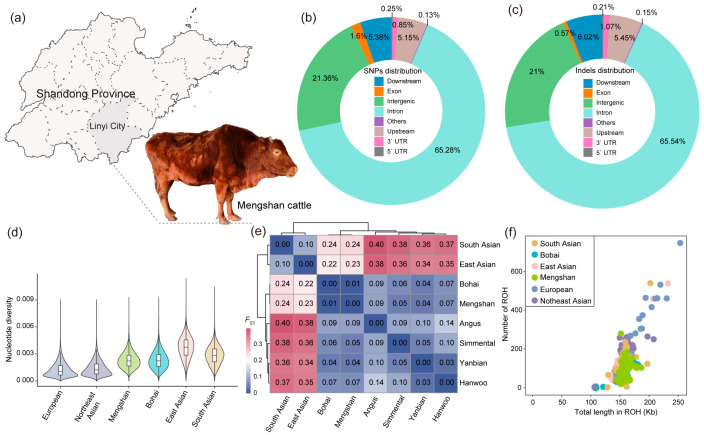
Genetic diversity of Mengshan cattle. (**a**) Main producing areas of Mengshan cattle. (**b**) Functional classification of the detected SNPs. (**c**) Functional classification of the detected Indels. (**d**) Violin plot of nucleotide diversity for each population. (**e**) Genetic distances estimated between each population by the *F*_ST_. (**f**) Total length and total number of ROHs per individual in each population.

To investigate the genomic features and selection signatures in Mengshan cattle, we sequenced 37 new whole-genome sequences (Table S1) of Mengshan cattle from Linyi City (Figure 1a) at the Novogene Bioinformatics Institute in Beijing, China. Additionally, we gathered 85 whole-genome sequences from previous studies for comparison [2]. These included 16 European taurine cattle (9 Angus, 7 Simmental), 22 Northeast Asian taurine cattle (13 Hanwoo, 9 Yanbian), 19 Chinese indicine cattle, 10 Bohai black cattle, and 15 Indian indicine cattle (Table S2).

### 2.2. Reads Mapping and Variant Calling

Clean reads from the 37 samples were mapped to the bovine reference assembly ARS-UCD1.2 using the Burrows–Wheeler Alignment MEM (BWA-MEM) (v0.7.13-r1126) with default parameters [14]. Duplicated reads were removed, and sequence alignment map (SAM) files were sorted and merged using SAMtool [15] before importing into Picard (Available online: http://broadinstitute.github.io/picard (accessed on 22 August 2023)). Variant calling for single-nucleotide polymorphisms (SNPs) and insertions/deletions (Indels) was performed using the Genome Analysis Toolkit 3.8 (GATK 3.8) [16]. SNPs were separated from the VCF files, with biallelic SNPs extracted. Filtering criteria for raw SNPs and Indels included QD < 2.0, FS > 200, and ReadPosRankSum < −20.0. Annotation of SNPs and Indels for each breed was conducted using SnpEff v3.0 with the latest *Bos taurus* reference genome [17].

### 2.3. Population Structure and Phylogenetic Analysis

Based on the previous classification of domestic cattle worldwide, we analyzed 85 domestic cattle with known population structures [2] and 37 Mengshan cattle with unknown pedigrees to assess the current population structure and phylogenetic relationships of Mengshan cattle. Population structure analysis and principal component analysis (PCA) were carried out using PLINK v1.9 [18] with the parameters (--indep-pairwise 50 5 0.2). ADMIXTURE [19] was employed for population structure prediction, setting kinship (*K*) from 2 to 3. PCA was performed using smartPCA of EIGENSOFT [20]. A phylogenetic tree was constructed from the analyzed dataset using PLINK’s distance matrix [18].

### 2.4. General Genomic Characteristics

Nucleotide diversity for each breed was assessed with a sliding window approach (50 kb windows, 20 kb steps) using VCFtools v0.1.16 [21]. Genetic differentiation between groups was measured by the fixation index (*F*_ST_) [22]. Runs of homozygosity (ROH) were detected using PLINK v1.9 [18] with parameters set to a minimum length of 100 kb, a scanning window size of 100 SNPs, a minimum density threshold of 200 SNPs, a large gap of 1000 kb, and a maximum number of heterozygous SNPs.

### 2.5. Detection of Selection Signals

The composite likelihood ratio (CLR) method was utilized to test for selection signatures in both Mengshan cattle and European taurine cattle. The CLR test involved splitting the entire genome into nonoverlapping 50 kb windows, which were then analyzed using SweepFinder2 v2.0 [23]. This approach allows for the detection of regions in the genome that have undergone selective pressure by comparing the likelihood of observed genetic variation under models with and without selection.

For comparisons between Mengshan cattle and Northeast Asian taurine cattle, we employed three key population genetic metrics: fixation index (*F*_ST_), genetic diversity (*θ*_π_ ratio), and the cross-population extended haplotype homozygosity test (XPEHH). *F*_ST_ and *θ*_π_ ratio analyses were performed in 50 kb windows with 20 kb steps using VCFtools v0.1.16 [21]. XPEHH statistics were calculated for each population pair using Selscan v1.1 [24]. In the interpretation of the above results, we identified putative selective sweeps by selecting the top 1% of the original scores from each method, which represent the most likely candidates for the regions under selection.

Candidate genes for positive selection were further defined as those supported by at least two of the methods mentioned above. To understand the biological significance of these candidate genes, we performed functional enrichment analysis using KOBAS 3.0 [25], with a *p* value of less than 0.05 indicating significant enrichment.

## 3. Results

### 3.1. Data Collection and Identification of Indels and SNPs

We generated genomic data from 37 samples, producing approximately 11.3 billion reads aligned to the ARS-UCD1.2 reference genome with an average mapping rate of 99.83%. The average depth of the reads was 16.27× (Table S1). After the filtering, we retained 55,305,564 biallelic SNPs and 5,138,989 Indels.

After we annotated the function of the polymorphic sites using SnpEff, the results showed that most of the SNPs were in intronic regions (65.28%), and exons accounted for 1.60% of the total SNPs, with 603,598 missense SNPs and 1,214,832 synonymous SNPs (Figure 1b and Table S3). The majority of the Indels was located in the intron regions (65.53%); the intergenic Indels accounted for 21.00%, including 2,632,124 insertions and 3,965,574 deletions (Figure 1c and Table S3).

### 3.2. Genomic Characteristics

The genetic variation patterns of the six cattle populations/breeds were analyzed by evaluating nucleotide diversity, genetic distance, and ROH. Nucleotide diversity analysis showed that current Mengshan cattle have higher genetic diversity compared to other commercial cattle breeds, suggesting a broader genetic base (Figure 1d). The genetic distances, estimated using *F*_ST_, ranged from 0.17 to 0.40, with Mengshan cattle and Bohai Black cattle showing the closest genetic relationship (Figure 1e). This close genetic distance implies a significant degree of similarity between these two breeds. The ROH analysis indicated that the total length of ROH in Mengshan cattle ranges from 130 to 200 Kb (Figure 1f). This measure provides insights into the extent of inbreeding and historical genetic changes within the population.

### 3.3. Population Differentiation and Genetic Structure

**Figure 2:**
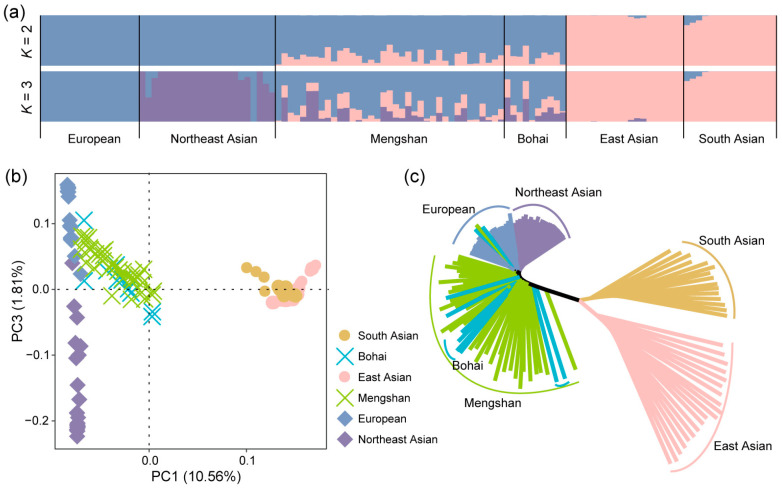
Population structure of Mengshan cattle. (**a**) Genetic structure of cattle populations using ADMIXTURE from K = 2 and K = 3. (**b**) Principal component analysis of cattle populations with PC1 against PC3. (**c**) Neighbor-joining tree of relationships among populations.

Based on the genomic SNP data, the ADMIXTURE, PCA, and neighbor-joining (NJ) tree methods were used to explore the population differentiation and phylogenetic relationships between Mengshan cattle and other cattle breeds. We used clustering models to predict ancestral populations and set *K* = 2 through *K* = 3 for ADMIXTURE analysis for all 122 samples. When *K* = 2, the cattle breeds were genetically separated from *B. taurus* and *Bos indicus* ancestry. At *K* = 3, we obtained the most reasonable biological explanation; current Mengshan cattle show obvious genetic characteristics, European taurine cattle ancestry is the main part, with a small number of indicine cattle and Northeast Asian taurine cattle ancestry (Figure 2a). This result highlights the significant influence of European taurine cattle on the genetic makeup of current Mengshan cattle, while also indicating some degree of genetic contribution from other sources. Further, the population composition of Bohai Black cattle showed similarities to Mengshan cattle, suggesting a close genetic relationship between these two breeds. The PCA analysis, which visualizes genetic relationships by plotting samples based on their principal components, and the NJ tree analysis, which constructs a phylogenetic tree based on genetic distances, both corroborated the findings from the ADMIXTURE analysis (Figure 2b,c).

### 3.4. Genetic Signature of Positive Selection in Mengshan Cattle

**Figure 3:**
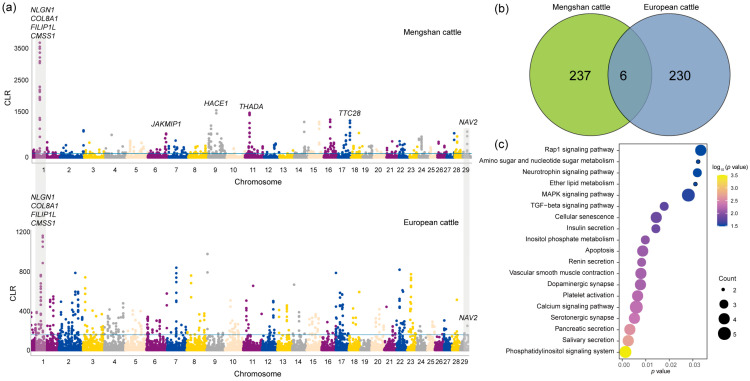
The signatures of positive selection in Mengshan cattle. (**a**) Manhattan plot of selective sweeps of Mengshan cattle and European taurine cattle by CLR method. (**b**) Genes shared by CLR methods in Mengshan cattle and European taurine cattle. (**c**) KEGG enrichment analysis results of 237 genes in Mengshan cattle.

Based on the results of the population structure of Mengshan cattle, we found that Mengshan cattle had most of the European taurine cattle ancestry. To identify the role of European taurine cattle ancestry in the genome of Mengshan cattle, the CLR method was used to detect the genome selection characteristics of Mengshan cattle and European taurine cattle populations. Outlier regions (the top 1%) identified with the CLR method were regarded as breed-specific candidate regions for analysis (Figure 3a, Tables S4 and S5). A total of six common genes were found, including *NAV2*, *NLGN1*, and *NOX3* genes associated with neurodevelopment, and *CMSS1*, *COL8A1*, and *FILIP1L* genes associated with muscle development and lipid metabolism (Figure 3b). In addition, 237 genes in Mengshan cattle were analyzed for KEGG enrichment, and we found that the results were related to insulin secretion, calcium signaling pathway, MAPK signaling pathway, and Dopaminergic synapse (Figure 3c, and Table S6).

**Figure 4:**
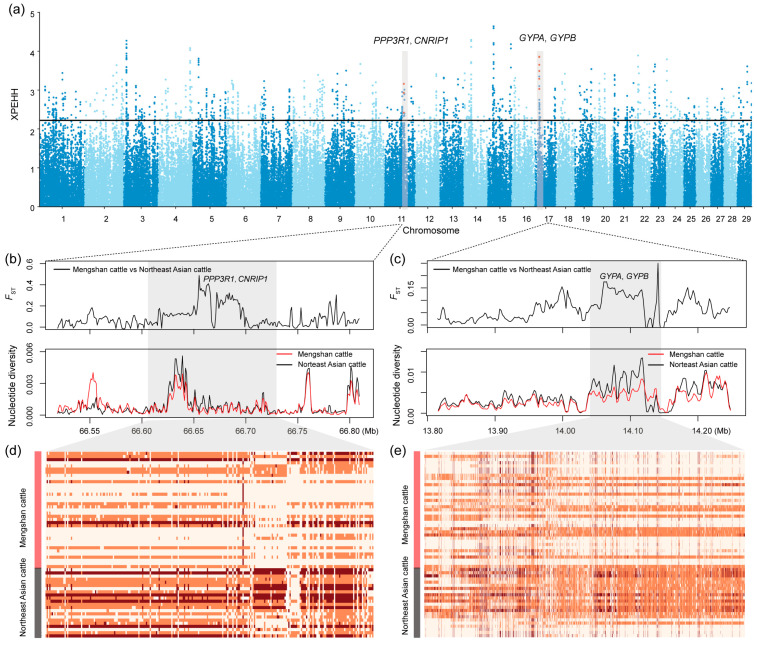
Selective signals between Mengshan cattle and Northeast Asian taurine cattle. (**a**) Manhattan plot of selective sweeps by XPEHH method. (**b**) *F*_ST_ and nucleotide diversity of *PPP3R1* and *CNRIP1* gene region. (**c**) *F*_ST_ and nucleotide diversity of *GYPA* and *GYPB* gene region. (**d**) Degree of haplotype sharing of *PPP3R1* and *CNRIP1* gene region. (**e**) Degree of haplotype sharing of *GYPA* and *GYPB* gene region.

Furthermore, we implemented three methods (*F*_ST_, *θ*_π_ ratio, XPEHH) to further elucidate the positive selection characteristics between Mengshan cattle and Northeast Asian taurine cattle (Figure 4a, Tables S7–S9). Each method provided unique insights into the regions of the genome under positive selection. Genomic regions identified as the top 1% by at least two of these methods were considered candidate regions for positive selection (Table S10). This multi-method approach increases the robustness of our identification of selection signatures. Among the candidate genes identified, we focused on those with functional importance in several biological areas, including immunity (*APPL2*, *CD48*, *CD84*, *CNRIP1*, *GYPA*, *GYPB*, *MUC6*, *NAALADL2*, *PLCG1*, *PRG3*, *SLAMF1*, and *SLAMF6*), growth traits (*ACAT1*, *CA10*, *GAB1*, *GLDN*, and *NUP98*), and meat quality (*CAPN14*, *CCDC3*, *DPYD*, *FOXP4*, *MAP2K1*, *MASP1*, *MTMR3*, *PPP3R1*, and *SLC43A3*). These genes may help Mengshan cattle adapt to local disease pressures and environmental challenges. Additionally, they reflect long-term selection for improved meat quality traits.

We compared haplotypes and identified two relatively long regions. These regions showed lower nucleotide diversity, indicating gene fixation in the population, and higher *F*_ST_ values and haplotype heatmaps, demonstrating significant differences between Mengshan cattle and Northeast Asian taurine cattle (Figure 4b–e). This suggests that these regions have likely undergone selective sweeps due to adaptation and targeted selection.

## 4. Discussion

Mengshan cattle, one of Shandong’s three major local breeds, play a pivotal role in the agricultural civilization of the southern mountainous areas of Shandong Province, bearing significant cultural value [9]. However, they are currently listed as an endangered genetic resource [10]. To date, research into the genetic makeup of the Mengshan cattle population remains sparse, underscoring the need for a more in-depth analysis of their genomic characteristics. Studies on genetic diversity and population structure are crucial for the evaluation of livestock genetic resources, understanding environmental adaptability, and the development and utilization of genetic resources [26,27]. Hence, we conducted whole-genome resequencing of 37 Mengshan cattle and selected representative cattle breeds for comparative analysis to explore the genetic diversity and population structure of Mengshan cattle.

Genetic diversity is a vital indicator of the richness of animal genetic resources in a region and holds significant importance for the conservation of animal genetic resources and the tailoring of breeding strategies. To assess the genetic diversity among Mengshan cattle, we evaluated nucleotide diversity, genetic distance, and ROH, analyzing the genetic variation patterns of six cattle populations/breeds. Compared to various commercial cattle breeds, Mengshan cattle exhibited higher nucleotide diversity. However, being primarily of taurine ancestry, their nucleotide diversity was still lower than that of indicine cattle, as is consistent with previous research [3,28]. The genetic distance of Mengshan cattle to other breeds ranged between 0.17 and 0.40, with the furthest distance from indicine cattle and the closest to the Bohai Black cattle, aligning with population structure analyses. The presence of long ROHs indicates inbreeding, while short ROHs reflect ancient ancestral influence. The ROH results suggest that Mengshan cattle have a lower degree of inbreeding and a higher degree of genetic diversity, with total ROH lengths ranging between 130 and 200 Kb, fewer in number and shorter in length compared to commercial cattle. In addition, at present, Mengshan cattle are mainly of European taurine cattle ancestry, with only a small number from Northeast Asian taurine, indicating that the conservation awareness of Mengshan cattle needs to be improved.

Given that Mengshan cattle are primarily of European taurine ancestry, to reveal the impact of European taurine ancestors on Mengshan cattle, we employed the CLR method to detect selected genes in Mengshan and European taurine cattle, and six genes were in strong selection. Genes related to neural development play crucial roles in training the brain for adaptive behavior essential for survival in changing environments [29,30]. The *NAV2* gene, influencing the development of the nervous system through all-trans retinoic acid (atRA), plays a significant role in mammalian neural development and is involved in the central perception of body fluid sodium levels and the regulation of salt intake behavior [31,32], which may be crucial for Mengshan cattle’s salt supplementation. The *NLGN1* gene, primarily expressed at excitatory synapses, where the dynamic modification of synaptic strength or plasticity is considered a cellular basis for adaptive behavior [33], is vital for Mengshan cattle’s adaptation to environmental changes. The *NOX3* gene, highly expressed in the inner ear, is important for hearing [34,35]. These genes play essential roles in Mengshan cattle’s continuous learning, formation of adaptive behaviors, and adaptation to environmental changes.

The Mengshan cattle are characterized by their compact physique and robust constitution, resulting in fine meat quality traits, which have been attributed to specific genes. *CMSS1* is associated with fatty acid metabolism, promoting fat deposition [36]. *COL8A1* is linked to the proliferation of muscle-derived satellite cells (MDSCs), facilitating muscle growth [37]. *FILIP1L* regulates collagen deposition and fat formation in muscles by influencing fibro-adipogenic progenitors (FAPs) [38]. These genes related to meat quality traits and fat deposition are consistent with the excellent meat quality features of Mengshan cattle. Additionally, KEGG enrichment analysis of the remaining 237 genes in Mengshan cattle revealed associations with pathways related to insulin secretion [39], calcium signaling [40], MAPK signaling pathways [41], and dopamine synaptic transmission [42], highlighting their importance in the economic traits and environmental adaptation of Mengshan cattle.

Subsequently, we conducted a comparative analysis between Mengshan cattle and Northeast Asian taurine cattle to identify advantageous genes specific to Mengshan cattle relative to those of Northeast Asian cattle. This study has identified several functional genes related to the economic traits of Mengshan cattle, which hold significant importance for the breeding and preservation of local breeds. Among the genes related to growth traits, *ACAT1*, *GAB1*, and *NUP98* are associated with feed efficiency and have been screened in experiments related to rumen expression and beef cattle weight gain [43,44,45]. *CA10* and *GLDN* are related to skeletal development and metabolism [4], with *CA10* involved in the dissolution of bone minerals and bone absorption [34] and *GLDN* involved in bone metabolism [46]. These genes contribute to the rapid growth of Mengshan cattle into robust individuals capable of adapting quickly to harsh environmental conditions.

Fat deposition affects meat quality and animal productivity, and several genes involved in fat metabolism were identified in this study. Among them, *CAPN14* is associated with seam fat and has been selected in multiple beef cattle breeds [47,48]. Through genome-wide association analysis and transcriptome data, *FOXP4* has been found to be associated with intramuscular fat content in Nellore and is involved in lipid metabolism [49]. *MAP2K1* is involved in lipid metabolism, with its protein expression levels being associated with the age of beef cattle, thus being useful for controlling the rate of fat deposition during growth [50]. Similarly, *SLC43A3* acts as a regulator of free fatty acid flux and plays a crucial role in fat metabolism [51]. Additionally, *MASP1* is associated with marbling in beef, enhancing the taste of beef [52]. *PPP3R1*, through its involvement in the MAPK signaling pathway, regulates meat tenderness [53,54,55]. These genes affect beef quality in various aspects, providing scientific evidence for the excellent meat quality of Mengshan cattle.

Mengshan cattle, a native Chinese breed, demonstrate remarkable resilience and environmental adaptability. Prolonged exposure to extensive rearing conditions has endowed Mengshan cattle with outstanding immune capabilities. This study identified several genes associated with immune responses that are under selection in Mengshan cattle. Among these, the APPL2 protein serves as an adaptor protein regulating both immune response and olfactory function [56], playing a crucial role in immune responses [57]. The *MUC6* gene is related to gastrointestinal parasite resistance [58,59]. *PLCG1* plays a vital role in the host’s response to ticks by regulating intracellular calcium ion concentrations [60]. The *PRG3* gene stimulates histamine biosynthesis and activates eosinophil formation to create a protective barrier [61], a mechanism that renders the tick attachment and feeding environment hostile, thus enhancing cattle’s resistance to parasites [62]. *GYPA* and *GYPB* are associated with human malaria and serve as invasion receptors for the malaria parasite in red blood cells [63,64], and their structural variations play a significant role in natural resistance to malaria [65,66]. Therefore, we speculate that these genes may have similar roles in Mengshan cattle, which is worthy of further study.

## 5. Conclusions

In conclusion, our study leveraged whole-genome sequencing to explore the population structure of current Mengshan cattle, elucidating its genetic diversity and conducting selective sweep analysis. Our findings underscore the predominant European taurine ancestry in Mengshan cattle, shedding light on their evolutionary history and genetic background. Moreover, we identified pivotal candidate genes associated with immunity, growth and development, meat quality traits, and neurological development, providing novel insights into the genetic basis of adaptive traits in this indigenous breed. These discoveries not only advance our knowledge of Mengshan cattle’s unique characteristics but also hold implications for the broader understanding of indigenous Chinese cattle breeds.

## Data Availability

The original data presented in the study are openly available in GenBank (BioProject accession number PRJNA1097992).

## Supplementary Materials

The following supporting information can be downloaded at https://www.mdpi.com/article/10.3390/genes15091113/s1, Table S1: Summary of sequencing statistics. Table S2: Summary of additional cattle sample information. Table S3: Functional annotation of the SNPs and Indels of Mengshan cattle by SnpEff. Table S4: A summary of genes from CLR in Mengshan cattle. Table S5: A summary of genes from CLR in European taurine cattle. Table S6: KEGG pathway of 237 genes in Mengshan cattle. Table S7: A summary of genes from *F*_ST_ in Mengshan cattle. Table S8: A summary of genes from *θ*_π_ ratio in Mengshan cattle. Table S9: A summary of genes from XPEHH in Mengshan cattle. Table S10: A summary of genes from at least two methods in Mengshan cattle.
